# Alcohol Induces Zebrafish Skeletal Muscle Atrophy through HMGB1/TLR4/NF-κB Signaling

**DOI:** 10.3390/life12081211

**Published:** 2022-08-10

**Authors:** Wei Wen, Chenchen Sun, Zhanglin Chen, Dong Yang, Zuoqiong Zhou, Xiyang Peng, Changfa Tang

**Affiliations:** Key Laboratory of Physical Fitness and Exercise Rehabilitation of Hunan Province, College of Physical Education, Hunan Normal University, Changsha 410012, China

**Keywords:** zebrafish, alcoholic myopathy, skeletal muscle atrophy, inflammatory

## Abstract

Excessive alcohol consumption can cause alcoholic myopathy, but the molecular mechanism is still unclear. In this study, zebrafish were exposed to 0.5% alcohol for eight weeks to investigate the effect of alcohol on skeletal muscle and its molecular mechanism. The results showed that the body length, body weight, cross-sectional area of the skeletal muscle fibers, Ucrit, and MO_2_max of the zebrafish were significantly decreased after alcohol exposure. The expression of markers of skeletal muscle atrophy and autophagy was increased, and the expression of P62 was significantly reduced. The content of ROS, the mRNA expression of *sod1* and *sod2*, and the protein expression of Nox2 were significantly increased. In addition, we found that the inflammatory factors Il1β and Tnfα were significantly enriched in skeletal muscle, and the expression of the HMGB1/TLR4/NF-κB signaling axis was also significantly increased. In summary, in this study, we established a zebrafish model of alcohol-induced skeletal muscle atrophy and further elucidated its pathogenesis.

## 1. Introduction

As the frequency of alcohol consumption gradually increases, the damage caused by excessive drinking to various organs in the body also continues to increase [1]. Skeletal muscle myopathy due to excessive alcohol ingestion, termed alcoholic myopathy [2], is characterized by progressive proximal weakness and skeletal muscle atrophy [3], which can significantly impair gait and mobility in this patient population. Alcoholic myopathy occurs in between 40 and 60% of individuals with chronic alcoholics [4,5], and the immediate cause is an imbalance between skeletal muscle protein synthesis and degradation [6,7,8,9]; however, the underlying cause is unclear. Therefore, extensive research is urgently needed to explore the underlying mechanisms of alcoholic myopathy and find effective interventions.

The regulation of protein degradation is mainly achieved by the ubiquitin‒proteasome system (UPS) and the autophagy‒lysosome system (ALS). Muscle RING finger 1 (MuRF1)/TRIM63 and muscle atrophy F-box (MAFbx), two E3 ligases in the UPS, are generally upregulated in alcoholic myopathy [2,4,10]. The ALS is also closely related to skeletal muscle atrophy [11,12], and autophagy levels were significantly increased in the skeletal muscle of chronic alcohol-drinking mice [6,13].

Inflammatory signaling leads to skeletal muscle atrophy by activating protein degradation [14,15,16,17,18]. The nuclear transcription factor NF-κB is a key regulator of inflammatory signaling pathways and promotes the expression of inflammatory factors [19,20]. NF-κB activity was significantly increased after the skeletal muscle of zebrafish was exposed to alcohol [21]. High mobility group box 1 (HMGB1), an essential mediator of chronic inflammation [22,23], activates NF-κB through Toll-like receptor 4 (TLR4) [24,25]. The expression of HMGB1 is significantly increased in patients with alcoholic liver disease who consume alcohol for long periods [26,27]. Therefore, we suspect that alcohol causes skeletal muscle atrophy by activating HMGB1/TLR4/NF-κB signaling.

Zebrafish, as a small and highly fecund vertebrate with a physiology and gene pool similar to those of humans, can be used to study the mechanism of action of ethanol in vivo [28,29]. The most significant advantage is the ease of administration, and water-soluble drugs such as ethanol can be directly mixed with water and subsequently absorbed by zebrafish [30]. In this study, we explore the molecular mechanisms of alcohol-induced skeletal muscle atrophy in zebrafish and provide new ideas for therapeutic targets of alcoholic myopathy.

## 2. Materials and Methods

### 2.1. Experimental Animals and Groups

The experimental animals were 8-month-old AB strain male zebrafish (*n* = 60) purchased from Wuhan Institute of Hydrobiology, Chinese Academy of Sciences, which were maintained under a light:dark cycle of 14 h:10 h. The operating procedures complied with the “Regulations on the Management and Use of Laboratory Animals of Hunan Normal University”. The experimental fish were divided into a control group (*n* = 30) and an alcohol exposure group (*n* = 30), which were reared in water or a 0.5 percent alcohol solution, respectively, for a total of 8 weeks of intervention. The control and alcohol exposure groups were fed fresh Artemia daily at 9:00 am, 1:00 pm, and 5:00 pm. The water or alcohol was changed every 24 h, and the alcohol concentration of the alcohol exposure group was measured daily. Hunan Normal University’s Laboratory Animal Ethics Committee approved this study (No. 2018-046).

### 2.2. Zebrafish Model Treated with Alcohol Exposure

Studies have shown that a 1% ethanol concentration can lead to the unnatural death of zebrafish, while zebrafish maintain a normal state in an ethanol concentration of 0.5% [31]. Therefore, a 0.5% ethanol concentration was selected in this experiment, which significantly changed the physiology of zebrafish without causing death.

### 2.3. Determination of Exercise Capacity and Maximal Oxygen Uptake

Ucrit is the maximum swimming speed achieved in the fish test protocol and it reflects the maximum ability of the fish to provide energy during continuous activity. It can be used as an evaluation index for fish’s swimming ability and metabolic performance [32]. Zebrafish’s motility and oxygen consumption were analyzed using a miniature swimming tunnel respirator (Loligo Systems, Viborg, Denmark). The specific test and calculation methods are as follows.

First, the body length and body weight of the zebrafish were measured and they fasted for 24 h. Second, the fasted zebrafish were transferred to the lane of the respirator for adaptive training at a rate of 0.8 body length per second (BL/S) for 2 h. After adaptation, the water speed in the respirator of the swimming lane was gradually increased according to the speed increment pattern of 1.35 BL/S every 7 min until the zebrafish reached the exhausted state (the standard of the exhausted state was that the zebrafish stopped at the water outlet of the swimming lane, ending 20 s above). Swimming lane respirator parameter settings during measurement were as follows: rinse for 90 s, wait for 30 s, and measure for 5 min. Finally, Ucrit was calculated based on the maximum swimming speed recorded by the instrument. The calculation formula was Ucrit = U_f_ + U_s_ × (T_f_/T_s_), where U_f_ is the swimming speed of the experimental fish when it is exhausted; U_s_ is the speed increment (1.35BL/S); T_f_ is the maximum swimming speed maintained before exhaustion time (min); T_s_ is the time interval (7 min) [32]. In order to eliminate the influence of zebrafish body length (BL) on swimming speed to a certain extent, the relative critical swimming speed (Ucrit-r) was used to calculate the maximum swimming speed of zebrafish, and the calculation formula was Ucrit-r = Ucrit/BL.

According to the real-time oxygen consumption MO_2_ (mmol/kg/h) recorded by the swimming lane respirator during the acceleration test, a graph was drawn between the acceleration nodes. Referring to the model equation calculated by Palstra, the oxygen consumption MO_2_ and velocity node equation were derived by regression analysis: MO_2_ = SMR + aU_BL_^2^ + bU_BL_, where SMR (basal metabolic rate, standard metabolic rate) represents the minimum oxygen consumption required to maintain metabolism in unfed zebrafish at rest; U_BL_ represents the ratio of real-time swimming speed to body length; a and b are constants. The maximal oxygen consumption of zebrafish is the value of oxygen consumption at the critical swimming speed.

### 2.4. HE Staining

Skeletal muscles were dehydrated, clarified in xylene, and embedded in paraffin. Serial sections of paraffin were cut using an RM2016 microtome (Leica, Wetzlar, Germany) and mounted on glass slides (Servicebio, Wuhan, China). H&E staining images were acquired using a NIKON DS-U3 camera control unit (Nikon, Tokyo, Japan).

### 2.5. DHE Staining

Fresh skeletal muscle tissue was placed on frozen slides, and ROS staining (DHE) solution (D7008, Sigma, diluted 1:500) was added to the marked area. ROS-positive areas were stained red with fluorescein. Positive areas were scored using ImageJ (version 5.0).

### 2.6. Total RNA Extraction and qRT-PCR

The skeletal muscle of zebrafish was collected from the upper region of the line connecting the zebrafish anterior vertebra and caudal vertebrae. RNA was extracted from zebrafish muscle tissue by homogenization in TRIzol solution, according to the manufacturer’s protocol (Thermo Fisher Scientific, Waltham, MA, USA). A reverse transcription system kit (Takara, Tokyo, Japan) was used to convert RNA to cDNA. SYBR green master mix (Thermo Fisher Scientific, Waltham, MA, USA) was used to perform real-time PCR. A real-time PCR system (CFX96, Bio-Rad, Hercules, CA, USA) was used to determine relative mRNA expression. Sangon Biotech synthesized primers for the detected genes and the reference gene gapdh. The relative mRNA expression was determined by using the 2-ΔΔCT method with the primers listed in Table S1.

### 2.7. Western Blotting

The skeletal muscle was lysed in RIPA buffer (High) (Solarbio, Wuhan, China) containing one mM phenylmethylsulfonyl fluoride (PFMS) and 1× protease phosphatase inhibitor (Solarbio, Wuhan, China). After quantification with a BCA protein quantification kit (Vazyme, Nanjing, China), 20 g of protein lysate was loaded onto an SDS‒PAGE gel. The PAGE gel was cut according to the molecular weight of the target protein, and the protein was transferred to a PVDF membrane after electrophoresis. The membranes were blocked with 5% nonfat milk at room temperature and incubated with primary antibody at 4 °C overnight. See Table S2 for specific information on the primary antibodies. After washing in PBST, the membranes were incubated with a goat anti-rabbit IgG-HRP secondary antibody (1:10,000, Absin, Shanghai, China). Proteins were detected using a gel imaging system (Tanon, Shanghai, China). Immunoreactive bands were visualized and quantified using ImageJ software.

### 2.8. Data Analysis

Statistics were analyzed with GraphPad Prism 8 (GraphPad Software, San Diego, CA, USA). All data were normally distributed and are presented as the mean ± SD of three independent experiments. Significance was determined using an unpaired-samples *t* test. *, **, *** represent *p* < 0.05, *p* < 0.01, and *p* < 0.001, respectively; ns represents *p* > 0.05, with no statistical significance.

## 3. Results

### 3.1. Alcohol Exposure Alters Zebrafish Appearance, Skeletal Muscle Morphology, and Exercise Capacity

**Figure 1:**
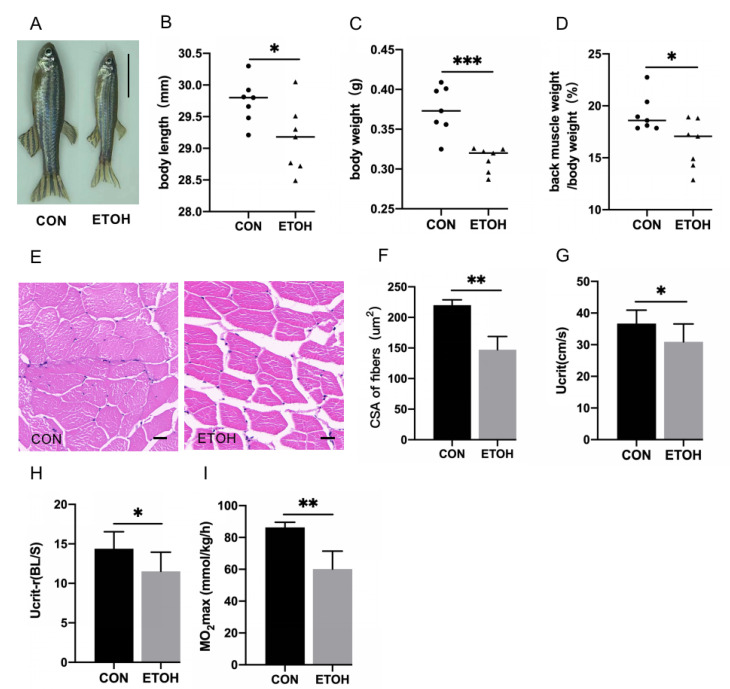
Morphology, skeletal muscle histology, and exercise capacity analysis of alcohol-induced zebrafish. (**A**) Comparison of appearance. Bar = 10 mm. (**B**) Body length (mm). (**C**) Body weight (g). (**D**) Back muscle proportion of body weight (%). (**E**) Hematoxylin–eosin (HE) staining. Bar = 20 µm. (**F**) Cross-sectional area (CSA) of muscle fibers (μm^2^). (**G**) Absolute critical swimming speed (cm/s). (**H**) Relative critical swimming speed (body length/second). (**I**) Maximal oxygen uptake (mmol/kg/h). *n* = 7. * *p* < 0.05, ** *p* < 0.01, *** *p* < 0.001.

After 8 weeks of 0.5% alcohol exposure, compared with those of the control group, the alcohol-exposed zebrafish’s body size (Figure 1A), body length (Figure 1B), body weight (Figure 1C), and back muscle weight/body weight (Figure 1D) were significantly decreased. The results of HE staining showed that the size and arrangement of skeletal muscle fibers in the alcohol exposure group were significantly changed (Figure 1E), and the cross-sectional area of muscle fibers was significantly reduced (Figure 1F). Zebrafish exercise capacity indices Ucrit, Ucrit-r, and MO_2_max were significantly lower in the alcohol exposure group than in the control group (Figure 1G–I).

### 3.2. Alcohol Exposure Activates the UPS and ALS of Zebrafish Skeletal Muscle

**Figure 2:**
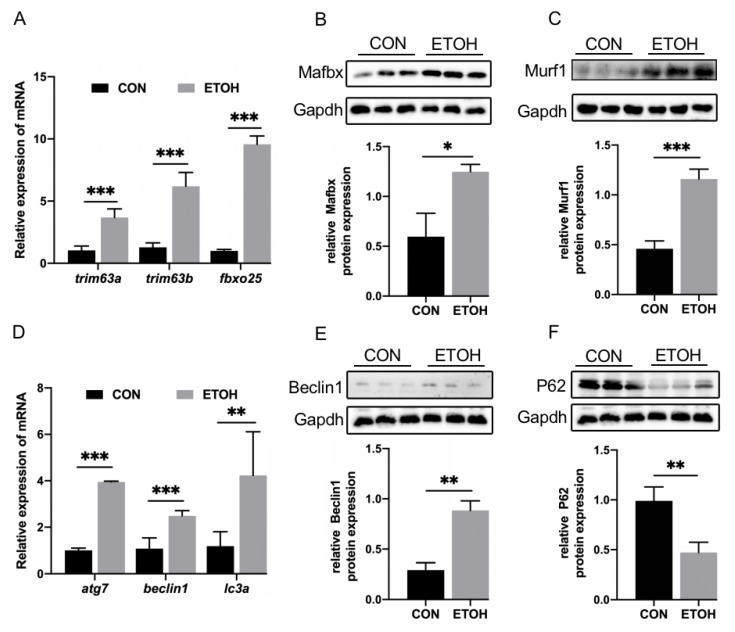
The effect of alcohol exposure on protein degradation in skeletal muscle. (**A**) Changes in the mRNA levels of *trim63a, trim63b*, and *fbxo25*. (**B**) Western blot analysis of the difference in Mafbx protein levels. (**C**) Protein expression level of Murf1. (**D**) mRNA expression of *atg7, beclin1,* and *lc3a*. (**E**) Western blot analysis of changes in Beclin1 levels. (**F**) Western blot analysis of changes in P62 levels. * *p* < 0.05, ** *p* < 0.01, and *** *p* < 0.001.

The main pathways of protein degradation are the UPS and ALS, which are involved in the degradation of more than 80% of proteins in cells [33]. The results showed that the mRNA levels of the UPS marker factors *trim63a*, *trim63b*, and *fbxo25* in the alcohol exposure group were significantly increased (Figure 2A). The protein levels of Mafbx and Murf1 were significantly increased (Figure 2B,C). The ALS mRNA levels of the marker factors *atg7*, *beclin1*, and *lc3a* were also significantly increased in the alcohol exposure group (Figure 2D), and the protein level of Beclin1 was significantly increased (Figure 2E), while the protein levels of the autophagy substrate P62 were decreased (Figure 2F).

### 3.3. Alcohol Exposure Increases ROS Generation and Redox System Dysregulation in Zebrafish Skeletal Muscle

**Figure 3:**
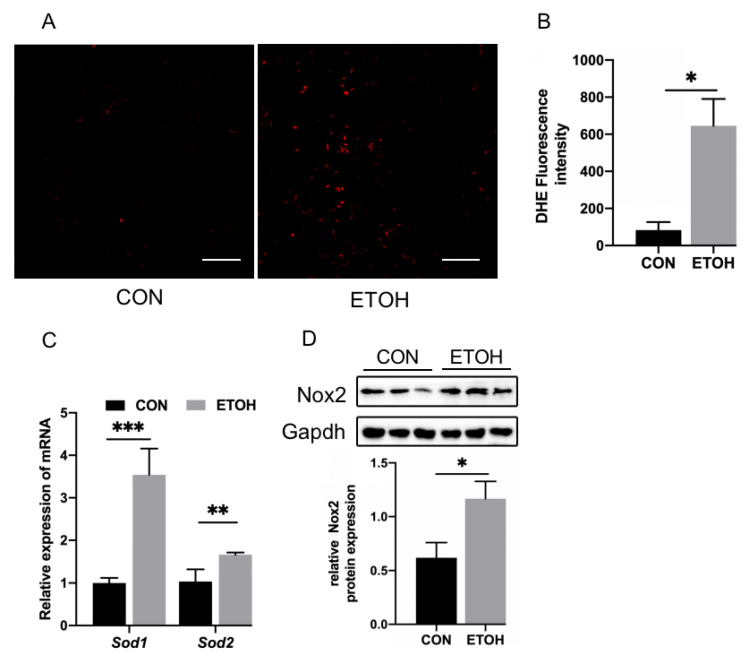
Zebrafish skeletal muscle ROS, oxidation, and antioxidant levels. (**A**) DHE staining of zebrafish skeletal muscle. Bar = 20 µm. (**B**) Quantitation of DHE staining. (**C**) mRNA levels of *sod1* and *sod2*. (**D**) Western blot analysis of Nox2 expression. * *p* < 0.05, ** *p* < 0.01, and *** *p* < 0.001.

We detected reactive oxygen species (ROS) in alcohol-exposed zebrafish skeletal muscle by DHE staining. The results showed that the content of ROS in the fibers of skeletal muscle in the alcohol exposure group was significantly increased (Figure 3A), and the fluorescence intensity of ROS was also significantly increased, as determined by ImageJ analysis (Figure 3B). The mRNA levels of the antioxidant enzymes *sod1* and *sod2* (Figure 3C) in the alcohol exposure group were significantly increased, and the protein expression of nicotinamide adenine dinucleotide phosphate oxidase 2 (Nox2) was significantly increased (Figure 3D).

### 3.4. Alcohol Exposure Activates Inflammation and the HMGB1/TLR4/NF-κB Signaling Pathway

**Figure 4:**
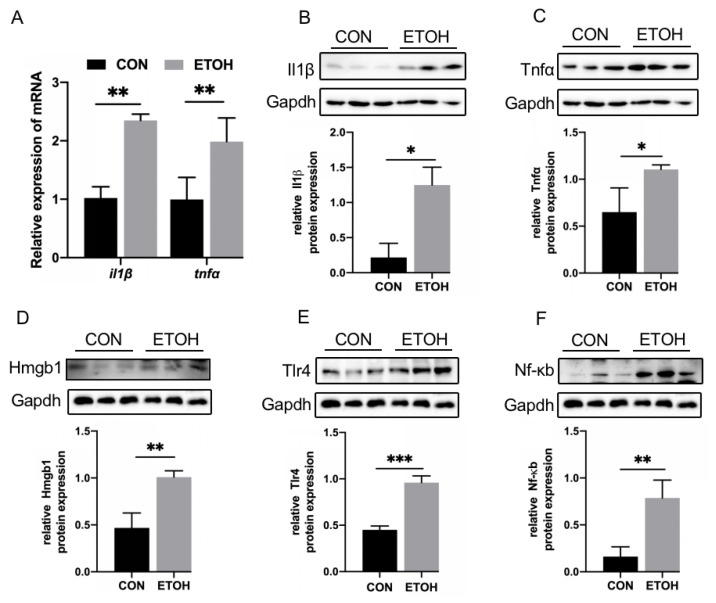
Inflammatory factors and HMGB1/TLR4/NF-κB expression in zebrafish skeletal muscle. (**A**) mRNA levels of *il1β* and *tnfα*. (**B**) Il1β protein expression level. (**C**) Tnfα protein expression level. (**D**) Hmgb1 protein expression level. (**E**) Tlr4 protein expression level. (**F**) Nf-κb protein expression level. * *p* < 0.05, ** *p* < 0.01, and *** *p* < 0.001.

Next, we tested alcohol-exposed zebrafish skeletal muscle for inflammatory factors and HMGB1/TLR4/NF-κB signaling. Compared with those in the control group, the mRNA levels of the inflammatory factors *il1β* and *tnfα* were significantly increased in the alcohol exposure group (Figure 4A). The expression of the Il1β and Tnfα proteins was also significantly increased (Figure 4B,C). Moreover, the protein levels of Hmgb1, Tlr4, and Nf-κb were significantly upregulated (Figure 4D–F).

## 4. Discussion

Prolonged and excessive alcohol consumption reduces skeletal muscle mass and impairs skeletal muscle function [34], but its pathogenesis remains understudied. In the present study, alcohol-exposed zebrafish had significantly reduced skeletal muscle size and exercise capacity, and the UPS and ALS were activated. Skeletal muscle inflammatory factor and ROS production was significantly increased, and HMGB1/TLR4/NF-κB signaling, which regulates inflammation, was also significantly increased. These results suggest that alcohol exposure causes skeletal muscle atrophy in zebrafish and that HMGB1/TLR4/NF-κB signaling may mediate alcohol-induced skeletal muscle atrophy.

Body weight, lean body mass, and skeletal muscle weight/whole body weight loss, as obvious markers of skeletal muscle atrophy, are commonly observed in models of alcohol-induced skeletal muscle atrophy [35,36,37]. Decreased muscle strength and increased perception of exercise fatigue have been reported in alcohol-fed mice [38]. In this experiment, zebrafish’s body weight, back muscle/body weight, and skeletal muscle fiber cross-sectional area were significantly reduced. Ucrit and MO_2_max values were significantly reduced after alcohol exposure, indicating that alcohol exposure leads to skeletal muscle atrophy and reduced exercise ability in zebrafish.

Impaired protein synthesis is an important cause of skeletal muscle atrophy. We experimentally validated the canonical signaling IGF1/PI3K/AKT pathway for protein synthesis. The experimental results showed that there was no significant change in the IGF1/PI3K/AKT signaling axis. Similar to the results of this experiment, alcohol reduces protein synthesis in alcohol-fed mice through non-IGF1/PI3K/AKT signaling [39]. A similar situation may exist in alcohol-exposed zebrafish, which is the focus of our next study. The UPS and ALS are the main pathways of protein degradation [40]. Autophagy markers were significantly enriched in alcohol-fed rats [41]. The mRNA levels of *murf1* and *mafbx* were also significantly increased in alcohol abuse patients [42]. In the present study, alcohol exposure increased UPS and ALS activity in zebrafish, similar to previous findings in rats [43]. This finding indicates that the protein degradation pathway dominated by the UPS and ALS was the direct cause of alcohol-induced skeletal muscle atrophy in zebrafish.

Inflammation is an essential cause of skeletal muscle atrophy [33,44]. NF-κB and the NF-κB-induced inflammatory factor TNFα can activate the transcription of *murf1* and *mafbx* [40,45,46]. The mRNA levels of *tnfα* and *il-6* were increased in the gastrocnemius muscle of adult rats fed alcohol [47]. In this study, the expression of the inflammatory factors Tnfα and Il1β was increased in the skeletal muscle of alcohol-exposed zebrafish, which may be the reason for the atrophy of the zebrafish’s skeletal muscle caused by alcohol exposure.

ROS production can be induced by the inflammatory factors Tnfα and Il1β [48,49], and TLR4 and TLR4/NF-KB are also closely related to ROS [50,51]. Nox is a common ROS-generating enzyme, and the expression of Nox2 is dependent on NF-κB activation [52]. In alcohol-exposed zebrafish’s skeletal muscle, Nox2 was activated, the ROS content was significantly increased, and the mRNA levels of *sod1* and *sod2* were elevated. This is similar to the experimental results and characteristics of alcohol consumption in rats and myotubes [9,53]. Although the expression levels of *sod1* and *sod2* were adaptively increased in this study, ROS levels were significantly increased in skeletal muscle, which may be due to the overactivation of Nox2, unbalancing the redox system and eventually generating a large amount of ROS in skeletal muscle.

HMGB1 is a DNA-binding protein, released extracellularly, that binds to TLR4, which triggers NF-κB signaling and induces an inflammatory response. The HMGB1 content was significantly increased in the serum of rats exposed to alcohol for a long period of time [54,55]. As a common receptor of HMGB1, TLR4 activation leads to the overexpression of MAFbx, MuRF1, and the autophagy markers LC3-II and P62 in C2C12 myotubes to induce skeletal muscle atrophy [17,56]. NF-κB, a downstream signal mediated by TLR4, is also closely associated with skeletal muscle atrophy [12,57]. HMGB1/TLR4/NF-KB is the primary signal regulating inflammation, and their protein expression was significantly increased in this experiment, indicating that they may be a potential regulatory mechanism in alcoholic myopathy.

In conclusion, we successfully constructed a model of alcohol-induced skeletal muscle atrophy in zebrafish, providing a model for the testing of treatments for human alcoholic myopathy. For the first time, in a zebrafish model, it was demonstrated that the process of alcohol-induced skeletal muscle atrophy is mediated by HMGB1/TLR4/NF-κB, which provides a reference for the treatment of alcoholic myopathy in humans.

## Data Availability

The data are available from the corresponding author.

## Supplementary Materials

The following supporting information can be downloaded at: https://www.mdpi.com/article/10.3390/life12081211/s1, Table S1: List of primers used for RT-qPCR; Table S2: List of primary antibodies used for Western blotting.
