# Supplementary material for: Alcohol Induces Zebrafish Skeletal Muscle Atrophy through HMGB1/TLR4/NF-κB Signaling
Source: Life (Basel). 2022 Aug 10;12(8):1211. doi: 10.3390/life12081211 (PMC9410481; doi:10.3390/life12081211)

Western blot original images of Fig.2

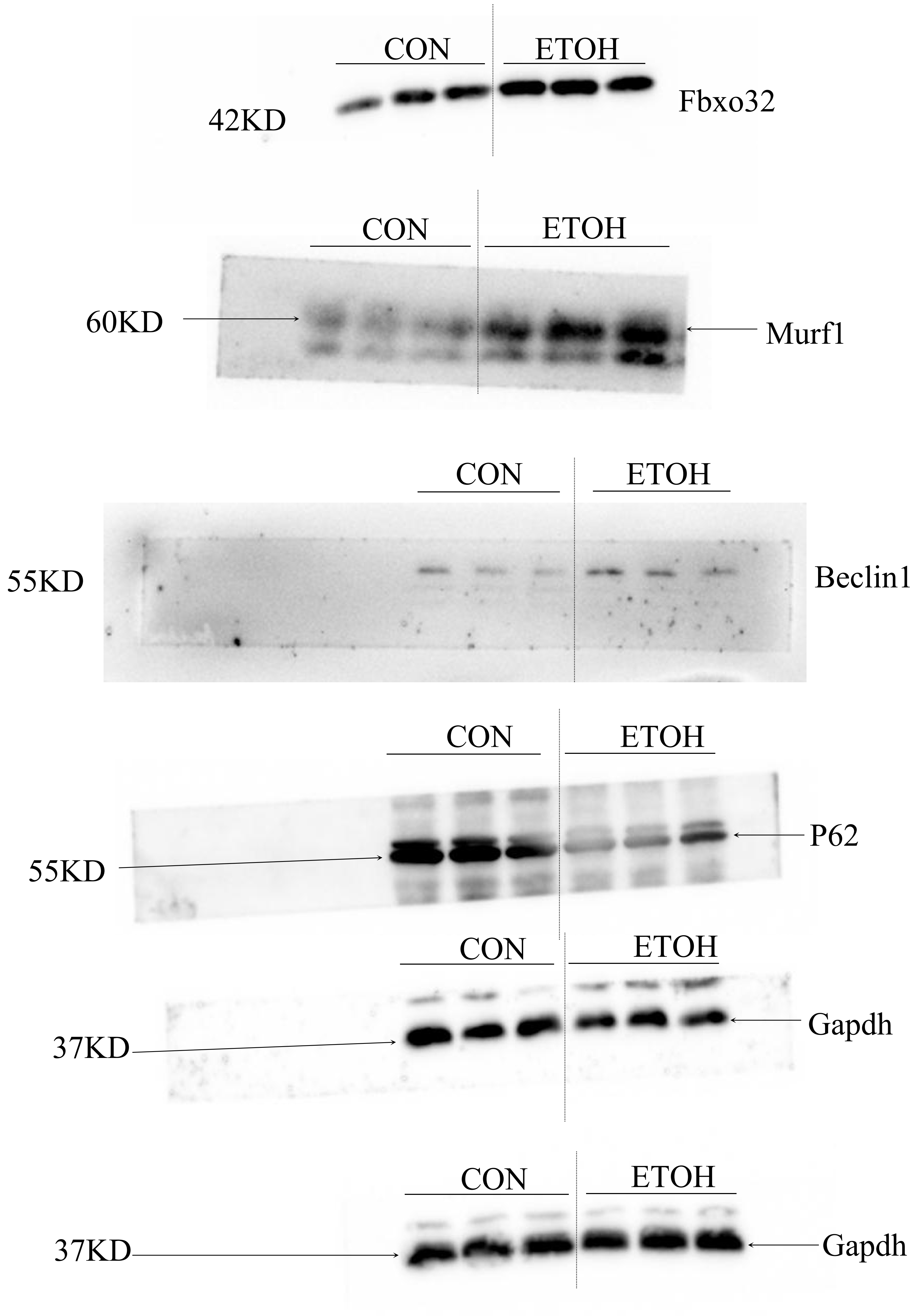

Western blot original images of Fig.3

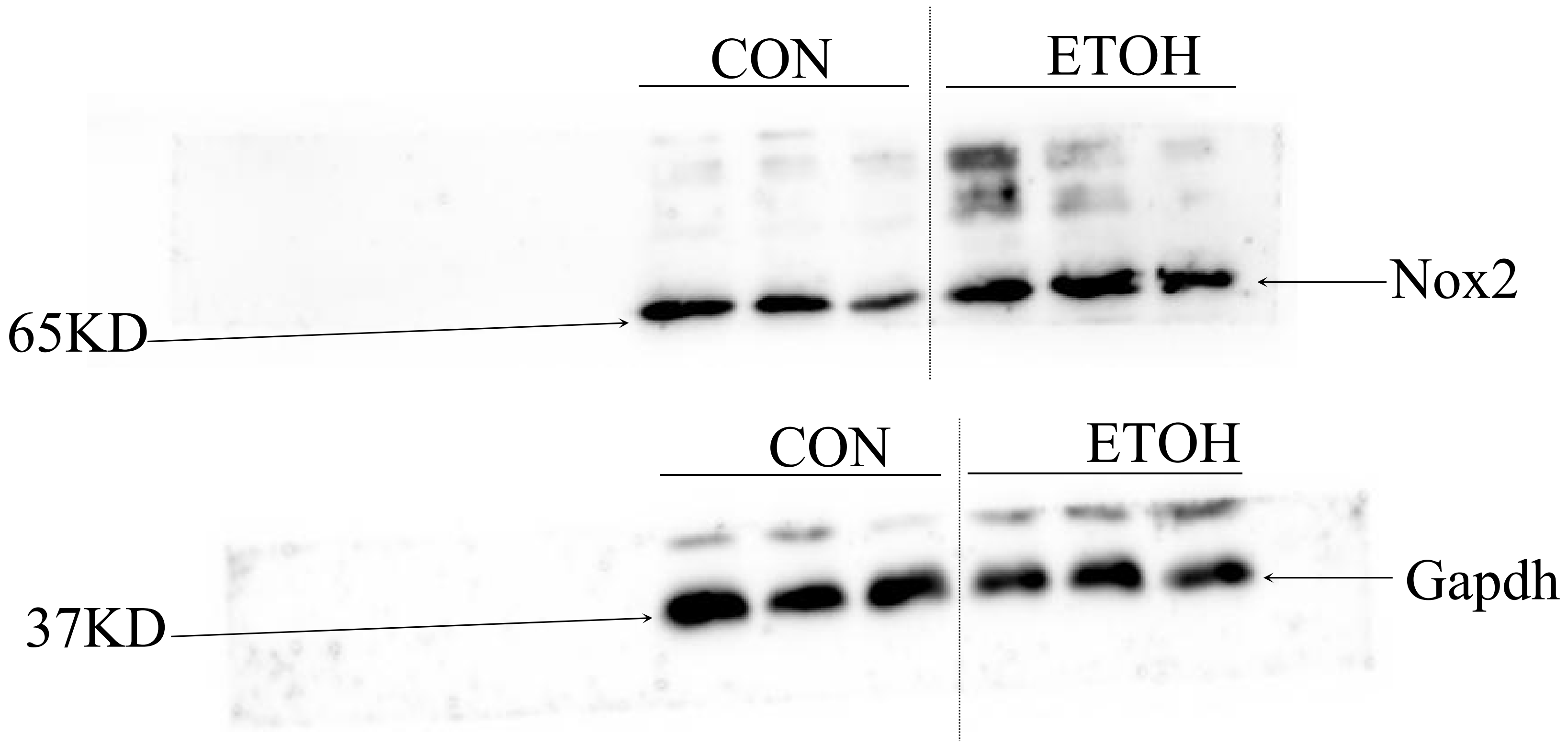

Western blot original images of Fig.4

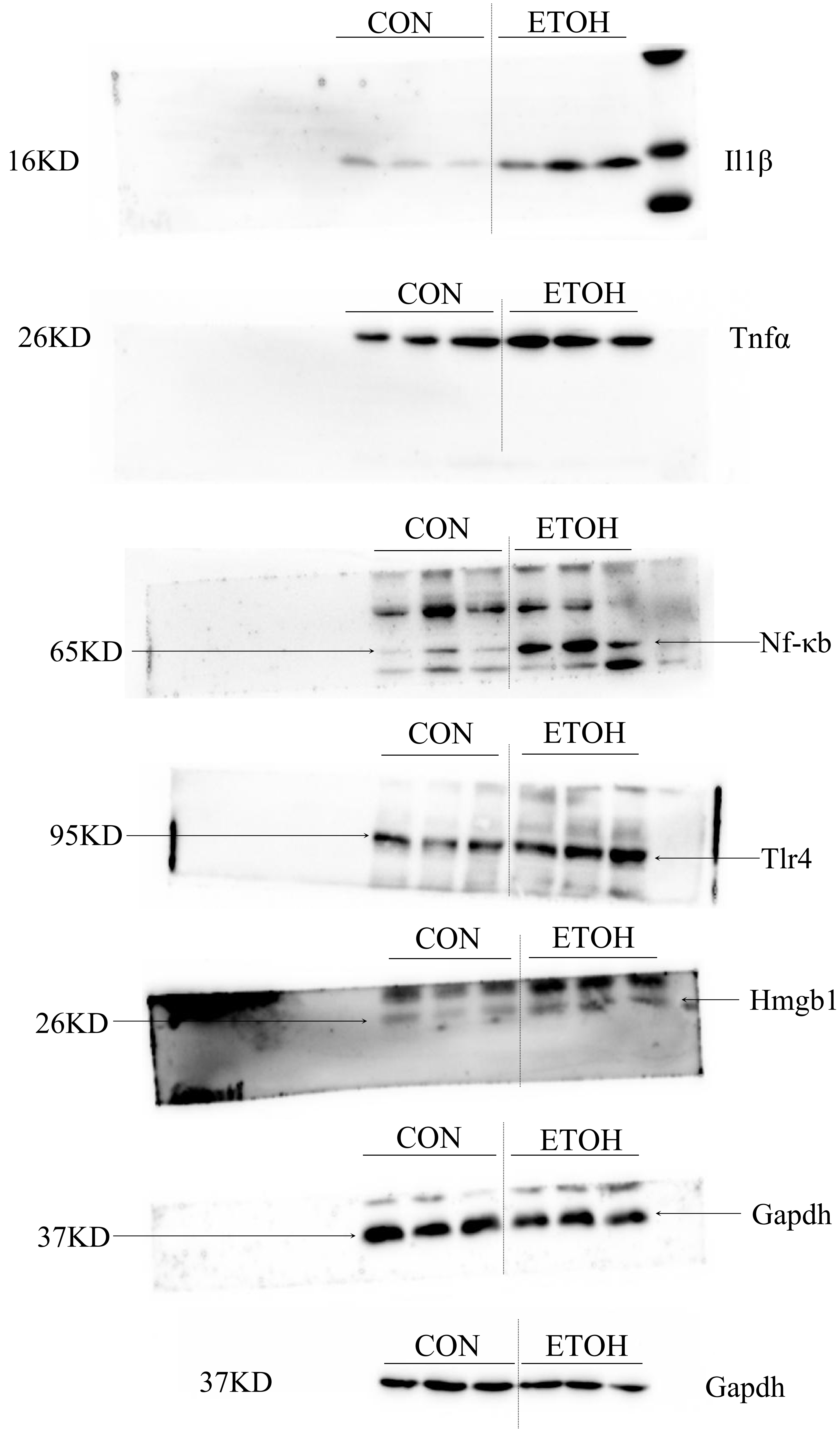

Supplement: Supplementary file 1 [file life-12-01211-s001.zip › Original image of Western blot.pdf]
